# The conservation genetics juggling act: integrating genetics and ecology, science and policy

**DOI:** 10.1111/eva.12337

**Published:** 2015-12-01

**Authors:** Susan M. Haig, Mark. P. Miller, Renee Bellinger, Hope M. Draheim, Dacey M. Mercer, Thomas D. Mullins

**Affiliations:** ^1^ U.S. Geological SurveyForest and Rangeland Ecosystem Science CenterCorvallisORUSA; ^2^ Department of Biology, Tropical Conservation Biology and Environmental ScienceUniversity of HawaiiHiloHIUSA; ^3^ Pacific States Marine Fisheries CommissionEagle Fish Genetics LaboratoryEagleIDUSA; ^4^ Hatfield Marine Science CenterOregon State UniversityNewportORUSA

**Keywords:** annual cycle, conservation genetics, cross‐seasonal interactions, effective population size, endangered species, inbreeding, migratory connectivity, pedigree analyses

## Abstract

The field of conservation genetics, when properly implemented, is a constant juggling act integrating molecular genetics, ecology, and demography with applied aspects concerning managing declining species or implementing conservation laws and policies. This young field has grown substantially since the 1980s following the development of polymerase chain reaction and now into the genomics era. Our laboratory has ‘grown up’ with the field, having worked on these issues for over three decades. Our multidisciplinary approach entails understanding the behavior and ecology of species as well as the underlying processes that contribute to genetic viability. Taking this holistic approach provides a comprehensive understanding of factors that influence species persistence and evolutionary potential while considering annual challenges that occur throughout their life cycle. As a federal laboratory, we are often addressing the needs of the U.S. Fish and Wildlife Service in their efforts to list, de‐list, or recover species. Nevertheless, there remains an overall communication gap between research geneticists and biologists who are charged with implementing their results. Therefore, we outline the need for a National Center for Small Population Biology to ameliorate this problem and provide organizations charged with making status decisions firmer ground from which to make their critical decisions.

## Introduction

The field of conservation genetics grew from studies of applied population genetics that started in the 1920s or earlier when scientists were concerned with conservation of germplasm resources for plants (reviewed in Allendorf et al. 2015). In 1950, Voipio identified key small‐population process and other genetic concerns in maintaining wildlife populations. However, major interest in developing the ideas related to small‐population conservation began in the late 1970s when efforts for conservation of biodiversity escalated, and especially in the 1980s as advances in computer technologies and molecular genetics made it possible to test and apply the theories being developed (e.g., Frankel 1974; Ralls et al. 1979; Ryman et al. 1981; Utter 1981, 1991).

The field gained widespread attention with a series of books written and/or edited by Michael Soulé and colleagues (Soulé and Wilcox 1980; Frankel and Soulé 1981; Soulé 1986, 1987; Soulé and Kohm 1989). Together, with additional volumes edited by Schonewald‐Cox et al. (1983) and Ralls and Ballou (1986), they brought together the scientists who formed the emerging field of conservation genetics; that is, those interested in processes that result in the conservation (or loss) of genetic diversity in small populations.

Contributors to these books considered novel ideas such as applying genetic factors in a conservation context, including measuring levels of heterozygosity in small populations, reducing inbreeding in captive or wild populations, and striving to maximize the effective size of a population. They also defined the concept of *population viability* by providing wildlife managers with stochastic genetic and demographic models developed to establish viability goals (e.g., a *viable* population is capable of maintaining itself without significant management over an agreed upon *time frame* with an agreed upon *degree of certainty*) rather than simple numeric population goals (e.g., set a population goal of 300 deer). While viability goals include more information about a population than numeric population goals, some seemingly specific viability goals [e.g., maintain 90% heterozygosity for 200 years (Soulé et al. 1986) or the 50/500 rule (Franklin 1980; Frankham et al. 2014)] put forth in these early works were more broadly suggested rather than specifically derived. Thus, they inappropriately tended to be adopted across all taxa regardless of life history traits, population history, or capacity for population regeneration. Regardless, the viability concepts and others proposed by the authors remain revolutionary and evolutionary for an emerging field of science (Jamieson and Allendorf 2012; Frankham et al. 2014).

As indicative of how rapidly this field has emerged, it took over two decades for it to be settled enough for conservation genetics textbooks to emerge [e.g., Frankham et al. 2002 (2010), Allendorf et al. (2015)]. The field continues to incorporate evolutionary concepts and the development of appropriate policies when addressing challenges to populations and species (Lankau et al. 2011; Sgrò et al. 2011; Angeloni et al. 2012; and Santamaria and Mèndez 2012).

In this paper, we describe three decades of the evolution of Susan Haig's Laboratory of Conservation Genetics during the time that the field of conservation biology emerged into a rigorous discipline. During those formative years of the field of conservation genetics, and as a graduate student under the mentorship of Lewis Oring, Haig worked to understand the interacting effects of mating systems, dispersal patterns, and genetic diversity for an endangered shorebird throughout its annual cycle (e.g., Haig 1987, 1992; Haig and Oring 1988a,b,c). She went on as a Smithsonian Postdoctoral Fellow at the National Zoo where she dovetailed this approach with the practical applications of small‐population processes to manage captive populations being developed by Jonathan Ballou (e.g., Haig et al. 1990). What emerged was an integrated view of how to approach small‐population research and conservation that included an understanding of the species biology throughout the annual cycle (i.e., full life cycle biology, migratory connectivity) as well as the genetic patterns that resulted from these behaviors. This integrated perspective formed a lifelong approach to science, conservation and this paper, and has hopefully also carried over to influence the students and employees that have worked in the laboratory over the years (Box 1).

Box 1 **Creating and maintaining careers**

*Reflections are those of Susan Haig*.

The USGS Conservation Genetics Laboratory in the Forest and Rangeland Ecosystem Science Center (FRESC) is a federal facility located on the Oregon State University campus. I have a faculty appointment so I can integrate teaching, graduate students, and postdocs into my research program if I wish or I can conduct research with USGS staff alone. I prefer a hybrid approach where I teach and direct more students than many of my federal colleagues but less than my academic colleagues. The FRESC Conservation Genetics Laboratory represents about half of my research program. The other component addresses population ecology of avian species, particularly shorebirds. I established these programs in 1994, and since then, significant changes have occurred in the life history strategies among laboratory members.

**Graduate students**

I have had 37 graduate students and postdocs to date, 31% were geneticists, 31% worked on a mix of genetics and population ecology, and 38% had no genetics aspect to their work. Happily, all are employed at their appropriate level. In addition, two laboratory technicians (authors Draheim and Mercer) completed M.S. degrees in our laboratory and laboratory techs (authors) Draheim and Bellinger have just completed Ph.D. and are starting postdocs. The only person who did not end up in their field of choice is a former Ph.D. student who had a child as a student. She has a very good job but not as an academic as she had hoped. The pressures of field work and tenure were not what she felt was healthy for her family.

Our laboratory has always enjoyed the presence of students or postdocs from various countries around the world including Brazil, Sweden, Denmark, China, Namibia, and the Netherlands. Regardless of their academic position, the international students have always broadened our perspective on approaches to science and life. We have also been involved in organizing international training courses on animal movement, migratory connectivity, and conservation genetics in the United States, Mexico, Brazil, Kenya, and Namibia. Securing funds for our travel and the students travel to a central location for classes is difficult, but it has resulted in a number of international students acquiring graduate student positions in our laboratory or another.

**Balance**

Just as I have learned to balance my work at the office and in the field, I am part of a growing movement to encourage work/life balance for employees. Despite the tremendous changes that have taken place since I was a graduate student, postdoc and young faculty member in the 1980–90s, it is clear that as a profession, we have work to do to address lifestyle demands or we risk losing the best and brightest researchers.

**Figure 1:**
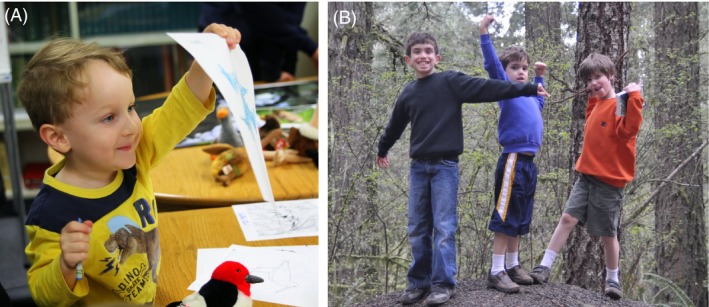
(A) Rohan Miller participates in our annual ‘Bring Your Kids to Work Day’. (B) Will, Ian, and Connor Mullins ‘help’ look for Spotted Owls during a laboratory field outing (photos by Susan Haig).

When I started 35 years ago, academia and the federal government were at the forefront of providing professional opportunities for women and minorities in the sciences. We all benefited from the addition of more diverse points of view at work, but it was a tremendous culture change: Women were finally getting more professional jobs but often at the cost of marriage and a family life. For example, I did not know any working women my age with Ph.D., who were married and had children. To this day, the very few women my age who have reached this seniority and have families relied on stay‐at‐home husbands when their children were young. Now as an employer, it is not easy balancing the needs of employees who must get work done with the needs of women and men who value having a family and careers. On one hand, over the past 21 years, we have been happy to celebrate the births of 12 babies among laboratory members, and an additional 13 children have parents who have worked in the laboratory (Fig. 1). But in only one case was a new baby born to a female student or postdoc. For that young woman, finishing her dissertation required an extra 18 months of funding (not easy for me to acquire) and a great deal of time away from school. The rest of the babies were born to technicians (one male, one female) and male postdocs who were not under the stress of finishing a degree. In all cases, the nonlaboratory parent also worked, and both parents wanted to spend significant amounts of time at home following the birth and as the child grew. While I do not have children, I am among others in my laboratory who care for elderly family members or those with special needs, and require schedule flexibility as well.

Thus, the challenge is to have open and ongoing communications, develop realistic schedules and clear expectations as we work to accommodate job schedules and caretaking needs. It is more easily attained with genetics personnel who often can work out more flexible schedules in a laboratory than with field personnel who need to spend significant amounts of time away from home. It can and must work as we encourage women interested in careers and families to participate in higher levels of science.

Here, we review our efforts to advance the field of conservation genetics for single species, primarily avian species, over a variety of temporal and spatial scales (Box 2). We consider advances made in the areas of (i) taxonomy, (ii) migratory connectivity, (iii) landscape genetics, (iv) small‐population biology, and (v) use of ancient DNA, often using our 25‐year study of the Northern Spotted Owl (*Strix occidentalis caurina*) as a primary example (Box 3). We further address closing the communication gap that often exists between geneticists and conservation practitioners who need information by proposing development of a National Center for Small Population Biology. Currently, the field of conservation genetics continues to grow at an exponential rate (Box 2, Haig and Avise 1996; Haig 1998; Haig et al. 2011, 2013), particularly with the advent of genomic approaches (Romanov et al. 2009; Angeloni et al. 2012). However, one aspect remains the same in single‐species studies: understanding the biology of a species throughout their life cycle and by considering information collected across a species’ range provides for maximally robust and useful conclusions.

Box 2 **Evolution of sampling and markers in conservation genetics studies**

Over the past 30 years, molecular data for avian studies have gone from identifying few (5–10) variable allozyme loci via starch gel electrophoresis to being able to screen tens of thousands of variable markers for hundreds of individuals in one lane on the Illumina high‐throughput platform. Concomitant with development of new molecular methods has been our ability to use simpler and less invasive sampling techniques.

Early allozyme studies on Piping Plovers benefitted greatly by being able to use proteins from red blood cells rather than extracting organs and sacrificing each bird (Haig and Oring 1988a). Haig travelled through Mexico with a liquid nitrogen container and a centrifuge that plugged into her truck. This work was further enhanced with the discovery that the pulp in growing feathers could also provide good results (Marsden and May 1984).

As with the entire field of molecular biology, discovery and development of the polymerase chain reaction (PCR, Mullis et al. 1986) revolutionized our ability to identify markers. Our first application of PCR was in DNA fingerprinting studies of Guam Rail, Micronesian Kingfisher, and Red‐cockaded Woodpecker pedigrees (Haig et al. 1994a,b, 1995). While a major step forward from allozymes given there were more markers to quantify (an avian‐specific issue), fingerprints were difficult to accurately score for an entire population unless every bird was run on the same autoradiogram with every other bird. We did this but it was time‐consuming and expensive.

**Figure 2:**
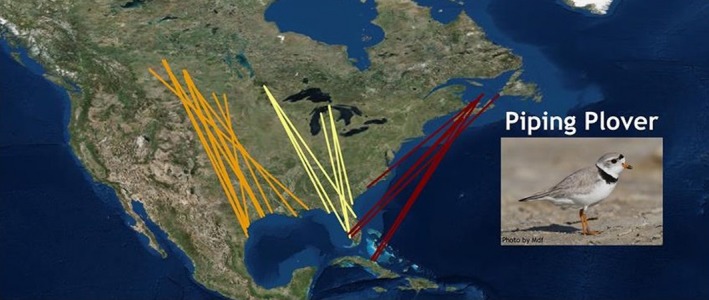
Migratory connectivity in the Piping Plover. Breeding locations for birds wintering in Bahamas confirmed via mtDNA markers to be in Atlantic coastal areas. Locations were further verified by resighting of color‐marked birds (data from Gratto‐Trevor et al. in press; figure produced from the Migratory Connectivity Project, www.migratoryconnectivityproject.org).

The next step in the evolution of markers was our use of random amplified polymorphic DNA (RAPDs). We used these dominant markers for screening population‐specific markers in migrating shorebirds and broad‐scale population studies of Red‐cockaded Woodpeckers and Northern Spotted Owls (Haig et al. 1993a, 1994c, 1996, 1997, 2001). Use of RAPDs was controversial in vertebrate population genetics studies because of issues related to repeatability (Ramos et al. 2008); however, they were quite popular with plant geneticists if they were not examining within‐population structure (Nybom and Bartish 2000; Bartish et al. 2007). We were extremely careful with our RAPD analyses and never had a problem with repeatability. Always fighting the issue of fewer variable loci present in birds, we used RAPDs until we were able to develop microsatellites for detailed population studies of Northern Spotted Owls (Box 3).

Box 3 **Third decade of spotted owl genetics**

**Figure 3:**
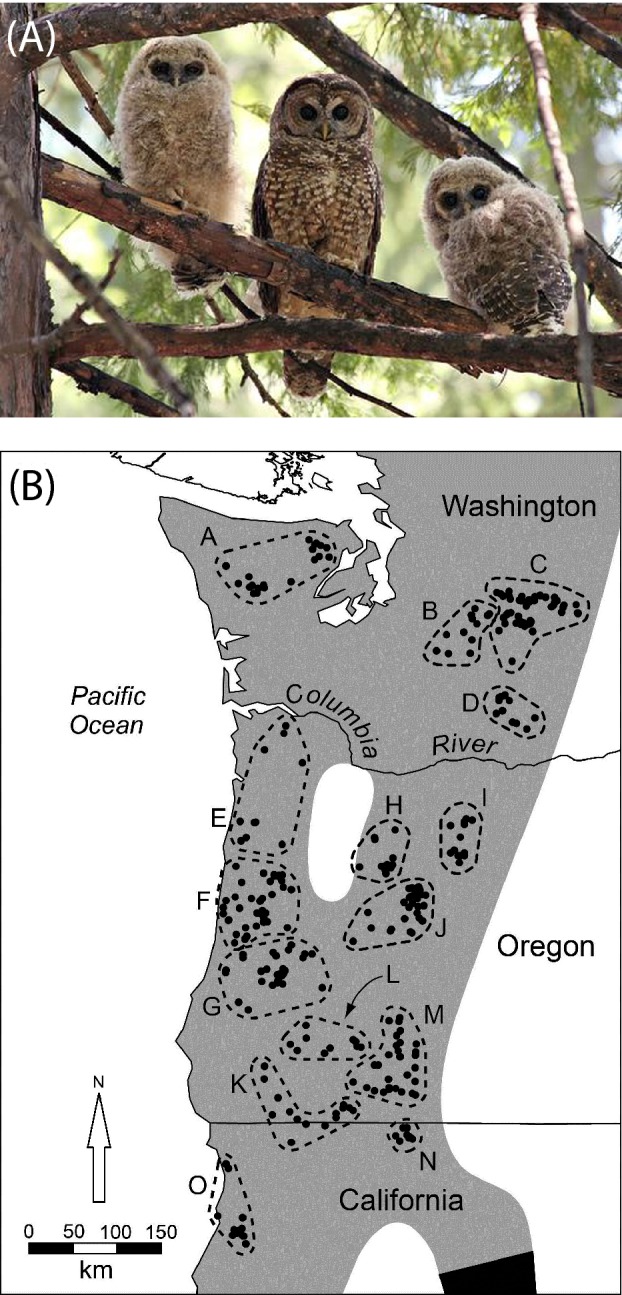
(A) Northern Spotted Owl female and two older chicks (photo by Sheila Whitmore), (B) Distribution of sample sites in the range of the Northern Spotted Owl (from Funk et al. 2010) (Box 3).

Few birds have been more controversial than the Northern Spotted Owl (Fig. 3, Forsman et al. 2011). They have represented the status of old growth forests in North America's Pacific Northwest for over three decades and as a result have evoked the ire of many logging communities while simultaneously rallying conservation groups (http://crosscut.com/2014/04/northwest-forest-plan-20-years-battles-obama/).

Perhaps the simplest but most critical molecular analyses needed for conservation of the Northern Spotted Owl was to define its taxonomic status (Fig. 3). There were millions of dollars of timber, jobs, and other resources riding on determining the limits of its range. Thus, it was imperative to determine if there were 1–3 species or subspecies to be considered for protection under the U.S. Endangered Species Act. In two studies using three markers (mtDNA, microsatellites, and RAPDs), we found agreement for three subspecies: Northern (*S. o. caurina*), California (*S. o. occidentalis*), and Mexican (*S. o. lucida*) with evidence for subspecies hybridization where taxa met geographically (Haig et al. 2001, 2004a,b).

The issue of intraspecific Northern‐California Spotted Owl hybrids complicated conservation action plans because the ESA only addresses issues for hybrids in captive situations (O'Brien and Mayr 1991). This became a bigger concern when we found evidence that Northern Spotted Owls were hybridizing with Barred Owls (*Strix varia*) that were quickly expanding their range into the Pacific Northwest. Not knowing how extensive this hybridization might be, we developed mtDNA, microsatellite, and AFLP markers to differentiate these taxa for use by law enforcement laboratories (Haig et al. 2004a,b; Funk et al. 2006, 2008a). Even after the markers were developed, there was a legal conundrum as to how to deal with a bird that looked like an ESA‐protected Northern Spotted Owl but genetically was a Barred Owl/Northern Spotted Owl hybrid. A little‐used clause in the ESA (section 4(e)) provided a potential solution (Haig and Allendorf 2006). This ‘similarity of appearance’ clause provides protection for species that are not listed but closely resemble an ESA‐listed species.

Understanding the genetic status of Northern Spotted Owls was the next important step. We began by taking a landscape genetics approach (Manel and Holdregger 2013) whereby we could examine the relationship between a random distribution of genes with a random distribution of geographic points across the range of the Northern Spotted Owl (Funk et al. 2008b). We did not find significant breaks in gene flow but we did find restrictions in gene flow in features such as the Cascade and Coast Range mountains as well as dry river valleys (Fig. 3). A closer investigation into restricted gene flow indicated that Northern Spotted Owls overall had likely undergone a significant recent population bottleneck (Funk et al. 2010). The results were the same when analyses were broken down by region (e.g., Cascade Mountains, Olympic peninsula, etc.) and local populations. The bottleneck signature was strongest for owls in the Washington Cascades, an area known to be experiencing a significant population decline (Forsman et al. 2011). In fact, when we compared our bottleneck results for local populations with population growth rates for the 14 demographic study areas monitored over the past 20+ years, there was a strong correlation between a significant population bottleneck and significant decline in lambda (population growth rate)(Funk et al. 2010). The one exception was a population that did not decline demographically but had experienced a significant bottleneck. Since identifying these bottlenecks, we continue to work to determine sources of inbreeding by examining pedigrees, juvenile dispersal patterns and other factors that may play a role in this important population process.

By the time, microsatellite studies were well underway, DNA could be extracted from almost anything it occurred in. This ranged from tail snips in salamanders (Miller et al. 2005) to extracting DNA from Red Tree Vole jaws obtained from Northern Spotted Owl fecal pellets (Bellinger et al. 2005). We were also able to extract DNA from California Condor (D'Elia 2015), Jabiru Stork (Lopes et al. 2010), and Least Tern (Draheim et al. 2010) museum tissue. Currently, we are using buccal swabs to collect thousands of samples from week‐old Red‐cockaded Woodpeckers. The ease of nondestructive sampling, including not needing to draw blood, has enabled us to acquire help from field technicians who require far less training to collect samples.

Progression toward using mtDNA sequencing, particularly in the control region, was a tremendous step forward in taxonomic assessments, basically becoming the work horse of taxonomy and phylogenetics (e.g., Haig et al. 2001, 2004a,b; Funk et al. 2007; Draheim et al. 2010; Lopes et al. 2010; Miller et al. 2010, 2012a,b, 2013a,b,c, 2015a,b; Mercer et al. 2013) . However, recent development of genomics techniques (discussed below) are steadily replacing traditional sequencing methods. The exponential increase in information yielded and our new ability to address questions of local adaptation and evolutionary potential, among many others, is phenomenal (Harrison et al. 2014; Prum et al. 2015). However, conservation genetics is a small field relative to the driving economic force of medicine in developing new technologies. Thus, even if current technologies would suffice in answering particular questions, conservation geneticists will need to adapt to the new technology as manufacturers will stop supporting current equipment in the future (McMahon et al. 2014).

Genomics refers to using multiple types of data generated by next‐generation sequencing such as transcriptome sequencing, SNPs (single nucleotide polymorphisms) typing, and whole‐genome methods (Lerner and Fleischer 2010; Angeloni et al. 2012; Zhang et al. 2014; Ogden 2015). The genomic approach provides the opportunity to better examine taxonomy, population biology, and conservation issues because of the number of loci used and speed at which loci can be identified and analyzed (Allendorf et al. 2010). Currently, the cost and time involved with whole‐genome sequencing results in fewer individuals being sampled than in traditional population studies. Thus, there is a tradeoff and questions need to be chosen carefully with this consideration in mind. There are also issues related to data management and bioinformatics with so much data being produced. This is in stark contrast to the past challenges of generating sufficient variable loci for resolution of informative population‐level studies.

There are few but an increasing number of genomic studies that clearly address conservation issues (McMahon et al. 2014). Not surprisingly, following delineation of the human genome, some of the first conservation applications were primate studies employing whole‐genome SNP data to infer demographic history and conservation (Prado‐Martinez et al. 2013; McManus et al. 2015; Osada et al. 2015). Others have included species as diverse as forest and savannah elephants (Rolandh et al. 2010), Lesser Kestrel (*Falco naumanni*; Wang et al. 2015) and Tibetan frogs (*Nanorana parkeri*; Sun et al. 2015).

We are just developing SNPs in our laboratory but can envision applying genomics in the future to all parameters of our conservation genetics studies, including estimating past and present demographic parameters, inbreeding, disease diagnosis, migratory connectivity and more. However, the primary impact will be the ability to sample a species’ genome at a level specific enough to identify regions responsible for local adaptation. This can be used for better implementation of conservation programs, particularly in evaluation of small populations in need of recovery.

## Delimiting taxa

Scientific debates over the definition of species and other taxonomic levels have occurred for hundreds of years (Wheeler and Meier 2000; Winker and Haig 2010; Gill 2014; Patten 2015; Toews 2015). This issue has become increasingly important because taxonomic uncertainty has ramifications that can result in protection (or denial of protection) under the IUCN Red List, Convention on Trade in Endangered Species (CITES), Migratory Bird Treaty Act, U.S. Endangered Species (ESA) Act, Canada's Species at Risk Act, and others (Haig and Allendorf 2006; Haig et al. 2006; Haig and D'Elia 2010). In the United States, this process becomes more complicated if ESA‐listed taxa hybridize with non‐ESA‐listed taxa (O'Brien and Mayr 1991; Haig and Allendorf 2006; Box 3) or if there are issues related to cryptic taxonomic patterns (Wagner et al. 2006; Funk et al. 2008a). Given uncertainty in criteria needed to define many of these issues, molecular data have become a cornerstone and key contributor to resolving many of these debates (Box 2 and 3; Zink 2004; Haig et al. 2006; Winker and Haig 2010; Lankau et al. 2011; Santamaria and Mèndez 2012).

In nonvolant species, dispersal can be low and the resulting divisions among taxa are easy to resolve as there is greater genetic divergence among populations. For example, endemic to the western United States is the *Arborimus* clade of arboreal *Phenacomys* voles with home ranges confined to a few trees. The short distances moved by these species appear to relate to easily defined differences among taxa *(*Bellinger et al. 2005; Miller et al. 2006). Likewise, Oregon Slender Salamanders (*Batrachoseps wrightii*) have a mean home range of less than 1 m, rendering them easy to genetically differentiate from other salamanders (Miller et al. 2005).

On the other hand, most birds can fly, even if nonmigratory, leading to greater likelihood for gene flow among populations and taxa. Thus, even with modern molecular markers, it can be difficult to find differences among avian populations, subspecies, etc. because differences may not exist or may not be as pronounced relative to less mobile taxa. Other influences such as the species concept debate, the financial costs of conservation, and the specific status assigned to each endangered species can affect conservation efforts and further illustrates the need for robust measures to define taxa. This is now going to be even more complicated as genomic data are added to this debate. Thus, while molecular data are now key in defining useful conservation units, there remains a need for scientific consensus regarding taxonomic definitions.

Molecular data used for taxonomic delineation can be useful in other conservation contexts aside from assessing uniqueness of taxa. We recently used ancient DNA extraction techniques on California Condor (*Gymnogyps californianus*) museum skins from the 1800s to obtain mitochondrial DNA sequences. These data allowed us to test the hypothesis that there may have been more than one species of North American condor in the past. Results indicated there was only one species (D'Elia 2015), suggesting that resource managers may be able to consider a wider geographic range for reintroductions of California Condors in the Pacific Northwest (D'Elia and Haig 2013).

Similarly, we are also examining museum tissue and blood samples for four described subspecies of the Micronesian Kingfisher (*Todiramphus cinnamominus*) from the Pacific islands of Pohnpei, Palau, Guam, and Ryukyu as a means of determining their true taxonomic relationships. The most immediate need for these analyses is to help plan reintroduction efforts for the Guam Kingfisher (*T. c. cinnamominus*). These birds went extinct in the wild in the mid‐1980s following introduction of the Brown Tree Snake (*Boiga irregularis*) to Guam (Savidge 1987). At that time, we designed a captive breeding program to conserve genetic diversity contributed from the remaining birds captured in the wild. This captive population was to be maintained until a suitable plan for reintroduction to the wild could be established (Haig and Ballou 1995; Haig et al. 1995). Then and now, the captive population would benefit from increased genetic diversity and overall numbers of kingfishers. Genetic diversity could potentially be increased by breeding the Guam birds with extant Micronesian Kingfishers from Palau (*T. c. pelewensis*) or Pohnpei (*T. c. reichenbacheii*) if they were are subspecies or closer. This option would only being considered if our taxonomic assessments indicated these populations were conspecifics.

The opposite issue existed in our analysis of the Double‐crested Cormorant (*Phalacrocorax auritus*; Mercer et al. 2013). Double‐crested Cormorants have been implicated in the decline of ESA‐listed Columbia River Basin salmonids, and there is pressure to implement lethal measures to control local cormorant populations. However, cormorants within some parts of their western North American range, especially Alaska and Mexico, have experienced significant declines, and the western breeding population was described as consisting of two subspecies distinct from the more abundant eastern cormorant subspecies. These subspecific designations had not been verified prior to our work, and additional information on population identity was needed to make informed management decisions. Mitochondrial and nuclear marker results indicated that there were significant differences between Alaska and western U.S. birds, so much so that genetic data supported recognition of an Alaskan subspecies distinct from other populations. Differentiation between western and eastern breeding populations was confirmed by differences in mitochondrial and nuclear DNA. Analyses also suggested a genetically unique population in the southwest portion of the range; however, permit issues precluded obtaining Mexican samples for analyses that were key to evaluating the presence of another subspecies in this region.

There will likely be no end to debates over taxonomic definitions. However, progress has been made in that agencies and conservation groups now consult with geneticists and rely on their results for key decisions. Yet there remains a critical gap to fill, because often geneticists are not familiar with the language of the ESA, particularly the definition of a species. Agencies have regulations and policy statements to provide more specific guidance than contained in the Act that may not be widely known. Likewise, individuals responsible for management decisions are often not familiar with interpretation of genetic data. Thus, providing a better explanation of the power and meaning of genetic results as they relate to ESA issues should be a goal for conservation geneticists.

## Small population biology

Our laboratory integrates a number of methods in an iterative manner to diagnose issues related to the recovery of small populations: field data, molecular analyses, pedigree analyses, and modeling (often viability modeling). An early opportunity occurred when Susan Haig was given the job of planning a captive recovery population for the Guam Rails (*Rallus owstoni*). They had just gone extinct in the wild as a result of the Brown Tree Snake introduction on Guam (Savidge 1987), and the remaining nine individuals were being maintained as a captive population. In the absence of modern molecular tools, Jonathan Ballou (Smithsonian Institution) and Susan Haig developed a series of pedigree‐related hypotheses to narrow the relatedness options among the captive population founders in order to provide pairing recommendations to the zoos. These hypotheses were tested using a pedigree analyses program called the ‘gene drop’ which is still the basis of most small population pedigree management (Haig et al. 1990; Haig and Ballou 2002; *PMx*—Lacy et al. 2012).

Once DNA fingerprinting became available, we were better able to quantify relatedness among the captive population founders (Haig et al. 1994a) and examine the effect our recommended pairings had on the population over the short time that the birds had been in captivity (Haig and Ballou 1995). Subsequently, we were able to adjust the breeding recommendations to improve the genetic and demographic status of the captive population. Although pedigree evaluation and management often is overlooked in wild populations (but see Haig et al. 1993a,b), this work illustrated that pedigrees can provide important information regarding the past, current, and potential structure of a wild population (Haig and Avise 1996; Haig and Ballou 2002). We are extending these ideas even further in our current work on Northern Spotted Owls and Red‐cockaded Woodpeckers (*Picoides borealis*, now *Leuconotopicus borealis*), which provide important breakthroughs that will greatly facilitate analyses of pedigrees in wild populations and increase the types of information that can be gleaned from them.

Modeling small populations and predicting their future viability began in the 1980s and continues to this day with spatially explicit models (SEPM) able to incorporate phenomenal amounts of data across a landscape (Beissinger et al. 2006; Chandler and Clark 2014). However, model results often are more general than a wildlife manager might require for their decision‐making processes. We addressed this issue in an effort to plan translocations for endangered Red‐cockaded Woodpeckers among old growth Longleaf Pine (*Pinus palustris*) stands across the southeastern United States (Haig et al. 1993a,b, 1994a,b, 1996). The woodpecker's decline is primarily due to forest fragmentation, which wildlife managers have tried to ameliorate by translocating birds from one forest to another.

As translocations began, questions remained regarding how many birds to move and which populations should be donors or recipients. We addressed this problem by examining phylogeography and population genetic structure across the species range to determine whether dispersal barriers were present, potentially limiting natural patterns of emigration and consequent gene flow. We did not find evidence of genetic discontinuities albeit there may not have been enough time for a genetic signal from newly separated populations. We did identify a significant relationship between genetic distance and geographic distance (i.e., isolation by distance) particularly from south to north as well as a larger body size in birds further north. We noted this might be part of the reason that translocations of birds north from Florida to Kentucky were unsuccessful. In this case, climate and selection may have played a part in translocation success. We further investigated the cooperative social system of woodpeckers to better understand what types of groups might need to be moved (i.e., families or whole social groups versus individuals; Haig et al. 1993a). We did not find evidence of extra‐pair fertilizations between helpers and breeders which helped understand effective size for various populations. Finally, we carried out viability modeling with the goal of determining the number of males and females needed to achieve specific population goals via translocations in specific areas each year over a 1‐, 5‐, and 10‐year period (Haig et al. 1993b). This approach provided managers with practical direction for their program as well as assurance that scientists can answer practical questions, not just academic or theoretical ones.

Our glimpse into historic population genetic trends of the endangered Least Tern (*Sternula antillarum*) illustrated a significant loss of genetic diversity in contemporary birds relative to pre‐1950 birds (Draheim et al. 2012). This information was helpful in illustrating the true status of the species when the ESA listing was being re‐considered by U.S. Fish and Wildlife Service. Our study was among the first times this sort of genetic loss concomitant to temporal decline had been documented for an avian species (also see Bellinger et al. 2003).

We undertook a similar approach examining historic population trends in California Condors (*Gymnogyps californianus*). In this case, doctoral student, Jesse D'Elia, collected tissue from every known California Condor museum skin (*n* = 67) and all 14 founders to the captive population, as well as pedigree information from the remaining extant birds (*n* = 404). Using 526 bp of the mtDNA control region, we found 18 haplotypes in the historical samples compared to three in the extant population—an astounding 80% decline in haplotype richness (D'Elia 2015). This is one of the largest population bottlenecks ever observed in nature. We further found there was no assorting of haplotypes by geographic region which is useful information as biologists develop breeding plans and consider options for reintroducing birds to the Pacific Northwest.

## Migratory connectivity

Migratory connectivity is the geographic connection of individuals and populations between one life cycle stage and another (Webster et al. 2002; Hostetler et al. 2015; Marra et al. 2015). The importance of understanding, researching, and applying this concept has always been a central focus of our laboratory and is best illustrated through our formation of the Migratory Connectivity Project (www.migratoryconnectivityproject.org) with Peter Marra and the Smithsonian Migratory Bird Center.

Traditionally, biologists interested in breeding biology and reproductive success worked at a single study site. In the 1980s, biologists studying waterfowl and shorebirds realized their study subjects spent 9+ months of the year in post/prebreeding sites and that many factors influencing birds breeding success and survival occurred during the months that they were not actively monitored (Myers 1981; Weller 1988). Attempts to investigate movement patterns by attaching colored leg bands and radio transmitters to many birds yielded limited results. Such markers need to be applied to many birds on the breeding grounds, and the same birds need to later be identified at their wintering location. This requirement poses numerous logistical challenges, ranging from the sample sizes need to obtain high probabilities of resights to the high cost of transmitters and travel to remote wintering areas. Genetic methodologies provide a powerful alternative to these approaches because once population‐specific markers are established, untold numbers of individuals can be sampled at any time of the annual cycle and their breeding origin can be established (Haig et al. 1997).

At the time, we first investigated this approach, and we used simple markers that could be quickly applied to hundreds, if not thousands, of shorebirds migrating from the high Arctic to southern wintering grounds and back (Box 2). Our approach was to screen for population‐specific markers, not just describe diversity among populations as is often done. We found population‐specific markers for Hudsonian Godwits (*Limosa haemastica*), Semipalmated Sandpipers (*Calidris pusilla*), and Dunlin (*Calidris alpina*). We also created criteria to identify winter origins of six other species. This research established the basis for identifying migratory connectivity using molecular data. Currently, selective choosing of SNPs (single nucleotide polymorphisms, see Box 2) will make identification of migratory paths even more specific and quantifiable.

Molecular data can be particularly useful in tracking migratory connectivity when birds move between continents over their annual cycle. A good example is our recent effort to define Dunlin (*Calidris alpina*) subspecies across Beringia in order to track the potential spread of bird flu across continents (Miller et al. 2015a). This type of study can be extremely difficult to implement in that sample collection required several years to complete and involved cooperation (and permits) among field biologists from Japan, South Korea, Russia, China, Canada, Alaska, and the lower 48 states. In the end, mtDNA markers were able to provide resolution of breeding site origin and migratory paths for birds that could be involved in disease transmission between continents. The study illustrates the massive scale of many current migratory connectivity studies, which requires cooperation among numerous individuals from different agencies, organizations, and countries to carry out all aspects of an investigation.

Even defining migratory connectivity within continents can be critical for conservation planning. For example, after identifying two Piping Plover subspecies (Haig and Oring 1988c; Miller et al. 2010) and over 30 years of trying to find the complete wintering distribution for the species (Haig and Oring 1985; Haig and Plissner 1993; Plissner and Haig 2000; Haig et al. 2005; Elliott‐Smith et al. 2009, 2015), a breakthrough occurred when biologists recently located birds in the Bahamas (Elliott‐Smith et al. 2009, 2015). Biologists then needed to identify the breeding origin of these birds in order to understand the importance of the Bahamas sites and determine whether the newly discovered winter sites were used by birds from a single breeding area or from across the species’ breeding range. Our mtDNA and microsatellite markers were able to place the birds as Atlantic Coast breeders (Gratto‐Trevor et al. in press; Fig. 1), thereby providing the important link identifying migratory connectivity for a critically endangered species.

Identifying annual or life cycle movements among archipelagoes further pinpoints potential areas of concern. For example, the Mariana Moorhen (*Gallinula chloropus guami*) occurs on four or more islands in the Marianas archipelago. New understanding of their seasonal movements and direction of juvenile dispersal is narrowing conservation foci to the most critical areas (Miller et al. 2015b).

## Phylogeography and landscape genetics

Increasing human population growth and land conversion due to human activities places stress on ecosystems. Formerly unaltered continuous habitats are presently fragmented into mosaics of varying habitat qualities, which creates within the landscape differential permeabilities to gene flow. Insights into population dynamics arise from identifying which factors influence degrees of population connectivity across these newly formed heterogeneous landscapes. The field of landscape genetics is formally described as an examination of the interaction between landscape features and evolutionary processes (Manel and Holdregger 2013). In our laboratory, we are interested in how the distribution of genes across a landscape varies with respect to the distribution of various geographic features and/or potential barriers to gene flow. This approach allowed us to test various hypotheses that evaluate genetic patterns relative to landscape patterns, particularly as they relate to anthropogenic versus natural barriers to dispersal or gene flow for various taxa. These comparisons are particularly powerful if temporal effects can be simultaneously considered (Miller and Haig 2010; Draheim et al. 2012).

Recently, we applied a landscape genetic approach to better understand the impact of habitat fragmentation on population connectivity among IUCN Red Listed Pfrimer's Parakeets (*Pyrrhura pfrimeri*). This species lives in the dry forests of north central Brazil's Cerrado (savannah) region which has undergone increasing habitat alternation due to deforestation since the 1970s (Miller et al. 2013b). We began using Landsat imagery to quantify the distribution and abundance of forest habitat in the region for three time periods: 1977, 1994, and 2008. We then used a novel measure of connection redundancy between populations to illustrate that genetic patterns in Pfrimer's Parakeet were most closely associated with forest conditions in 1977, indicating a 35+ year time lag between deforestation and contemporary genetic structure. Given continued deforestation in the region, genetic structure patterns are only expected to become stronger in the future. We are currently evaluating the future repercussions of continued deforestation in this system using spatially explicit models developed with HexSim (www.HexSim.net; Schumaker et al. 2014) to better understand the genetic and demographic outlook for Pfrimer's Parakeet.

Landscape genetic studies tend to examine patterns at a finer scale (e.g., dispersal distances of an individual) than phylogeographic studies, which investigate processes at greater temporal or spatial scales (e.g., phylogenetic breaks). Both are important approaches to understanding the underlying processes that impact spatial genetic structure of species. In a comparative study, we examined the spatial distribution of genetic variation in several species .(representing different taxonomic groups) with similar geographic distributions across Pacific Northwest forests (Miller and Haig 2010). Our objective was to explore which historical factors (e.g., glaciation and habitat alteration) best explained the observed genetic structure patterns of Northern Spotted Owls, Red Tree Voles (*Arborimus longicaudus*), Southern Torrent Salamanders (*Rhyacotriton variegatus*), and Western White Pine (*Pinus monticola*). Results differed by taxa: Genetic distances and diversity for Northern Spotted Owls and Western White Pine were greater in southern versus northern locales while genetic distances were greater in northern versus southern regions for Red Tree Voles and Southern Torrent Salamanders. Subsequent analyses suggested that historical factors such as range expansion, rather than anthropogenic factors, better explained some of these patterns. These types of analyses are important to perform prior to attributing human‐based activities to genetic structure patterns within species.

## Bridging the gap between scientists and managers

Much has been written defining the field of conservation genetics, and within each paper, there is a call for ‘bridging the gap’ between scientists who generate molecular data and conservation practitioners who use the data in a listing or status assessment or to establish recovery criteria and plan recovery actions. Most current species listing and recovery decisions require some form of molecular analyses to define taxa, assess status, and plan recovery; hence, there is a need: (i) for the molecular data and (ii) for agency personnel and organizations to understand how it applies to their listing and recovery information needs. These listing decisions by USFWS are not trivial. For example, the decision to list a species under the ESA, or even formal consideration of whether or not to list a species, can influence the management of recreational and economic activities on millions of acres of federal lands (USDA and BLM 1994, https://federalregister.gov/a/2015-12948). The cost for recovery of a listed bird species averages over $5 million per year (Gratwicke et al. 2012). Yet the personnel making decisions often do not have the appropriate training to interpret, let alone defend or refute the genetic implications of their decisions. This has become more serious as industry now hires molecular geneticists to refute listings. Thus, cases will be more fairly debated if there is equal representation on both sides of the discussion.

Another, albeit usually unspoken, issue is that the types of investigations required for listing and recovery decisions are often not prized or rewarded in academia. While exceptions exist, most single‐species conservation genetics work does not involve projects that result in publications accepted by the highest rated journals. Thus, the studies do not receive the attention they should in many laboratories. Furthermore, researchers need to realize that the best listing and recovery decisions are made by integrating results from a number of data sets (e.g., molecular, pedigree, demographic, PVA, etc.). However, few scientists are trained to collect, carry out analyses on, and interpret this diversity of information, let alone put it in an ESA, IUCN, or other policy context. Ultimately, the field of small population biology will evolve to be stronger and more effective for solving critical questions in conservation if we treat it as a multidisciplinary field unto its own (as the zoo community does) and not just any dataset that happens to deal with small populations. Thus, we need a focus on this integrated field itself.

### Solutions

The issues outlined above all call for a more focused, integrative, and informed approach to assessing species taxonomy or status. There are numerous short‐fixes that could be implemented such as short courses for conservation practitioners, more conservation genetics courses being taught at the undergraduate and graduate level, and sabbaticals for academics in decision‐making organizations such as USFWS endangered species offices. However, the contentious and serious nature of today's endangered species issues calls for a more significant solution.

Therefore, out of the same national and international need to develop focused expertise as was called for in the formation of the National Wildlife Health Laboratory (http://www.nwhc.usgs.gov/), National Wildlife Forensic Laboratory (http://www.fws.gov/lab/) and National Wetlands Laboratory (http://www.nwrc.usgs.gov/), development of a National Center for Small Population Biology would serve a similar purpose and would make major strides in bridging the gap between scientists and the decision‐makers who need to implement policies based on their results. Establishment of this Center would insure USFWS, and other partners were provided with the best information and latest technology to address an issue.

### Objectives

A National Center for Small Population Biology would provide scientific expertise to USFWS, and other organizations tasked with making decisions regarding species of concern. Approaches would include providing data and expertise in interpreting data from molecular, demographic, population modeling, viability modeling, and pedigree analyses. The peer‐reviewed products produced would provide assurance to partners that their population assessments have been undertaken with the greatest degree of scientific rigor possible as well as with careful attention toward specific needs for listing and recovery planning. Thus, the Center would strive to achieve the following objectives:

1. Provide agencies and organizations such as USFWS and IUCN with the most objective, comprehensive, and appropriate analyses from which they can make policy decisions regarding listing or recovering species at risk.
2. Provide recovery teams with expertise to help them appropriately design quantitative recovery objectives.
3. Provide training to agency, conservation practitioners, and decision‐makers in small population biology.
4. Provide opportunities for molecular population geneticists, particularly graduate students and postdocs, to learn about the management and policy applications of their work.
5. Provide guidance on data collection and management for species at risk.
6. Provide a receptacle for samples and data related to species at risk.
7. Advance the field of small population biology.

### Approach

A National Center for Small Population Biology would be a federal institution with a scientific advisory board of nonfederal experts from the fields of molecular genetics, pedigree analyses, population modeling, and endangered species law. Bringing this specific expertise under one umbrella group (perhaps virtual) would insure accuracy in results, consistency across plans, appropriate interpretation of law and policy, and integration between research and nonresearch biologists. The Center would be a place where participating scientists could either carry out analyses themselves or review analyses for organizations such as USFWS, rendering scientific judgement on whether they were valid, if they saw problems, etc. Ultimately, it would save conservation organizations time, money, and will ensure the data are the robust, appropriate for the questions being asked, and do not require additional expert interpretation. Giving an organization like USFWS, for example, a voice from the Center that could speak to the nuances of genetics, demography, the ESA, etc. would cut short and make clearer debates about the scientific merits of particular listing and recovery decisions. Under the current system, agencies and organizations hoping to find appropriate geneticists may need to perform time‐consuming searches when an issue arises, having a ready source of experts that could design and/or carry out research or consult about a species at risk would greatly facilitate this process. Development of the Center would not preclude other scientists from carrying out molecular or other types of research to address listing and recovery type issues. Rather, it would insure USFWS and other organizations always had the appropriate resources or consultants to carry out or interpret studies in the manner in which they needed.

### Molecular analyses

Increasingly, ESA and similar listings require molecular analyses to determine taxonomic identification, population structure, or population status (Box 3). This trend will only increase as managers and review groups require a more comprehensive information package to assess the conservation needs of a species. The rapidly evolving use of genomics can address those issues (Romanov et al. 2009; Haig et al. 2011). Further, integration of stable isotope data to identify north and south breeding locations while molecular data identify longitudinal patterns provide even more accurate location information (Clegg et al. 2003). Decision‐makers often do not have training to interpret genetic results provided to them by researchers nor do they know how to evaluate the strength of a molecular proposal submitted to them. This aspect of the Center would alleviate this problem.

### Demographic analyses

Demographic analyses are key to developing effective recovery plans and monitoring strategies (e.g., Forsman et al. 2011). Often field data are collected for years, yet formal analyses are not carried out because the data were not collected in a way that supports scientifically rigorous analysis, or the expertise is not available to apply emerging analytical and modeling techniques. Center demographers could design and carry out demographic analyses as well as integrate genetic and/or pedigree information into their models for a more comprehensive perspective.

### Pedigree analyses

Few people carry out pedigree analyses on wild populations (Haig and Ballou 2002). Even fewer combine molecular and modeling‐type pedigree analyses, yet they are critical for developing captive breeding, translocation, and reintroduction efforts (e.g., California Condor, Red‐cockaded Woodpecker). Center scientists would combine molecular, demographic, and pedigree analyses for severely threatened species to understand their status and improve their management in the wild and captivity.

### Population modeling

Population modeling, including Bayesian networks and spatially explicit models, can be extremely valuable in setting recovery objectives, informing listing decisions, and evaluating competing management strategies even when faced with uncertainty or incomplete datasets (McCracken et al. 2013; Pierson et al. 2015). Combining molecular and demographic data into these efforts would be an important aspect of building robust models to assess progress toward recovery.

### Database management

Many multifaceted endangered species programs have significant problems designing and collecting field data on a multisite, multiyear basis. The Center would provide this important service by helping organize, curate, and store population‐level data.

### Cryogenic sample repository (Sample ark)

Similar to data management, there is often a need to catalog tissue from genetic studies of species at risk following their analyses. The scientific and monetary value of these samples is incalculable. The majority of these samples could not be replaced and may represent the only historical snapshot of past diversity levels of these species. Thus, we can maintain a ‘Sample Ark’ which will only become more important as climatic and anthropological effects place increasing pressures on species of interest.

### Policy and legal expertise

Understanding and being conversant in the language and interpretation of laws and policies, such as the ESA and IUCN listing criteria, is essential in preparing listing packages and recovery plans. Integrating appropriate terms in genetic and demographic approaches to address some of the legal needs is not always obvious to a scientist. Center law and policy scholars could provide expertise to insure models address legal concerns such as critical habitat as well as biological issues.

### Outcome

The National Center for Small Population Biology would provide a unique and integrative bridge to USFWS, IUCN and other partners charged with assessment and recovery of species at risk. This tight connection will provide rigorous science directly applicable to the issues at hand and better integration of research findings into decision making. This would also have the added benefit of using these case studies to push the forefront of scientific approaches to small population research, management, and conservation.

A similar approach to the Center has evolved in the zoo community via formation of the IUCN Conservation Breeding Specialist Group (CBSG: www.cbsg.org) and the American Zoo and Aquarium Association Population Management Center (PMC: www.aza.org) at the Lincoln Park Zoo. These programs, run by overlapping personnel, train zoo personnel to manage the demographics and gene pools of single species located across many zoos. CBSG or PMC personnel carry out their own research as well as travel the world meeting with groups of zoo personnel involved with a particular species. Over the course of a few days, these scientists carry out genetic and demographic analyses needed to meet recovery goals while zoo personnel advise them on the reality of choosing various individuals to breed, transfer, etc. These programs have been phenomenally successful in managing captive and reintroduced species and as a result also have produced the most cutting edge research on small population biology management in the world. While the Center we are proposing primarily involves wild animals which likely are more complex to manage, the CBSG/PMC model is an excellent guide.

## Summary

In many ways, our approach to single‐species conservation genetics studies has not changed since Susan Haig's initial work on Piping Plovers in the 1980s. We continue to use extensive sampling to define taxonomy and population structure across the species range, understand migratory connectivity of populations, and consider behavioral and ecological factors in final syntheses of the data. However, the molecular methods and bioinformatics tools we now apply are more complex, leading to more specific, accurate, and useful conclusions. An important component of this brave new world is the ability to bridge the gap between genetics and population ecology to provide better information for status assessments and recovery planning. Development of a National Center for Small Population Biology will help bridge that gap.
